# Bacterial pneumonia and its associated factors in children from a developing country: A prospective cohort study

**DOI:** 10.1371/journal.pone.0228056

**Published:** 2020-02-14

**Authors:** Anna Marie Nathan, Cindy Shuan Ju Teh, Kartini Abdul Jabar, Boon Teong Teoh, Anithaa Tangaperumal, Caroline Westerhout, Rafdzah Zaki, Kah Peng Eg, Surendran Thavagnanam, Jessie Anne de Bruyne

**Affiliations:** 1 Department of Paediatrics, University Malaya Paediatric, Kuala Lumpur, Malaysia; 2 Child Health Research Group, University Malaya, Kuala Lumpur, Malaysia; 3 Department of Medical Microbiology, University Malaya, Kuala Lumpur, Malaysia; 4 Department of Tropical Infectious Diseases Research and Education Centre (TIDREC), University of Malaya, Kuala Lumpur, Malaysia; 5 Department of Biomedical Imaging, University Malaya Medical Centre, Kuala Lumpur, Malaysia; 6 Centre for Epidemiology and Evidence-Based Practice, Department of Social & Preventive Medicine, Faculty of Medicine, Kuala Lumpur, Malaysia; Public Health England, UNITED KINGDOM

## Abstract

**Introduction:**

Pneumonia in children is a common disease yet determining its aetiology remains elusive.

**Objectives:**

To determine the a) aetiology, b) factors associated with bacterial pneumonia and c) association between co-infections (bacteria + virus) and severity of disease, in children admitted with severe pneumonia.

**Methods:**

A prospective cohort study involving children aged 1-month to 5-years admitted with very severe pneumonia, as per the WHO definition, over 2 years. Induced sputum and blood obtained within 24 hrs of admission were examined via PCR, immunofluorescence and culture to detect 17 bacteria/viruses. A designated radiologist read the chest radiographs.

**Results:**

Three hundred patients with a mean (SD) age of 14 (±15) months old were recruited. Significant pathogens were detected in 62% of patients (n = 186). Viruses alone were detected in 23.7% (n = 71) with rhinovirus (31%), human metapneumovirus (HMP) [22.5%] and respiratory syncytial virus (RSV) [16.9%] being the commonest. Bacteria alone was detected in 25% (n = 75) with *Haemophilus influenzae* (29.3%), *Staphylococcus aureus* (24%) and *Streptococcus pneumoniae* (22.7%) being the commonest. Co-infections were seen in 13.3% (n = 40) of patients. Male gender (AdjOR 1.84 [95% CI 1.10, 3.05]) and presence of crepitations (AdjOR 2.27 [95% CI 1.12, 4.60]) were associated with bacterial infection. C-reactive protein (CRP) [p = 0.007]) was significantly higher in patients with co-infections but duration of hospitalization (p = 0.77) and requirement for supplemental respiratory support (p = 0.26) were not associated with co-infection.

**Conclusions:**

Bacteria remain an important cause of very severe pneumonia in developing countries with one in four children admitted isolating bacteria alone. Male gender and presence of crepitations were significantly associated with bacterial aetiology. Co-infection was associated with a higher CRP but no other parameters of severe clinical illness.

## Background

Pneumonia is a common disease across all economic strata, especially in children less than 5-years-old. While in developed countries its incidence is about 0.05 episodes/ child/year, in developing countries it is 0.22 episodes/ child/year and remains a common cause for unscheduled health care visits and hospitalisation[1–3]. Although childhood deaths from pneumonia have reduced significantly, pneumonia continues to afflict young children, especially from low- and middle-income countries (LMIC)[2]. Furthermore, severe pneumonia may result in long term complications like bronchiectasis which persist to adulthood and present as Chronic Obstructive Lung Disease[4,5]. Reduction in mortality and morbidity is dependent on timely and accurate treatment of these infections[6]. Therefore, being aware of the aetiology of pneumonia is crucial for successful management of the disease and planning of preventive measures such as immunisation hence impacting overall health of the young.

In an old study done in 1986, where lung aspirates were used to detect bacteria in children with severe pneumonia from LMIC, 62% had bacteria detected, mainly *Streptococcus pneumoniae* and *Haemophilus influenzae b* (Hib), while viruses were only present in 23%[7]. However, much has changed since then, with the introduction of Hib vaccine and the conjugate pneumococcal vaccine, where there are reports of reduction in infections attributed to these vaccine-preventable organisms[8–10]. In most LMIC, the Hib vaccine has already been included in their national immunisation programme. However, this is not the case for the pneumococcal vaccine, where even in Malaysia, this has yet to be incorporated into the national immunisation programme.

Yet, what is ironic about this age-old disease is that there is no gold standard to define bacterial pneumonia and no point-of-care test to differentiate between viral and bacterial pneumonia. Determining the aetiology of pneumonia remains difficult in children who cannot produce good quality sputum for culture.[11] Hence the reliance on molecular methods like polymerase chain reaction (PCR) and enzyme immunoassays (EIA) on blood and nasal secretions, and recently nucleic acid amplification test (NAAT) [12]to determine the true aetiology of pneumonia. These methods are expensive and have limited diagnostic accuracy as they are too sensitive and lack specificity[13,14].

The aims of this current study were to determine the a) aetiology of pneumonia, b) factors associated with bacterial pneumonia and c) association between co-infections and severity of disease, in children admitted with severe pneumonia.

## Methodology

### Study setting

This study took place in University Malaya Medical Centre (UMMC), a government-funded teaching hospital in Kuala Lumpur, Malaysia. This hospital has a maximum bed occupancy of 1650 and serves an urban population of more than 1 million in the district of Petaling Jaya. It has a separate children’s block which has 6 Paediatric wards, a Paediatric Intensive Care Unit (PICU), a Neonatal Intensive Care unit and outpatient clinics. UMMC also has a dedicated Paediatric Emergency Unit, the first in Malaysia.

### Study design and ethical approval

This is a prospective study conducted over 2 years, by the Paediatric Respiratory department in UMMC. Our local institutional ethics committee University Malaya Medical Centre Ethics committee (MEC IDNO 20146–336) approved this study. Informed signed consent was obtained from parents and all patient information was anonymised.

### Study population

This study included children aged 1-month to 5-years-old, who were admitted from 1^st^ October 2014 till 31^st^ October 2016, with very severe pneumonia as per the WHO definition[15]. Children who had doctor diagnosed asthma or recurrent wheezing of childhood (more than 2 episodes), refused blood taking, had symptoms > 7 days, were unable to come for follow-up, had chronic disease or had no chest radiograph performed, were excluded. Patients were recruited via convenient sampling from the paediatric wards.

### Study definitions

Very severe pneumonia was defined as history of cough and/or shortness of breath with examination findings of age-defined tachypnoea (defined as respiratory rate >60/min for infants <2 months; >50/min for infants 2–12 months; >40/min for children >12–60 months; and >30/min for children >60–144 months) and either one of the following: recessions, saturation < 92% on air, poor feeding or lethargy[15].

Life- threatening pneumonia was defined as that requiring either invasive or non-invasive respiratory support, PICU care or illness resulting in death.

Abnormal chest radiograph supportive of pneumonia was defined as the presence of either focal or diffuse infiltrates, silhouette sign, pleural effusion or air bronchogram[16].

Previous lung infection was defined as any previous history of a lower respiratory tract infection (LRTI), with the presence of shortness of breath during that illness. Exposure to environmental tobacco smoke was defined as presence of a family member living in the same household who smokes.

Defining a true bacterial infection based on the detection of pathogens alone is insufficient. Therefore using a conglomeration of symptoms, signs and investigation results to define aetiology of pneumonia was used in this study, modified from the Bacterial Pneumonia Score.[17] Bacterial aetiology was presumed if there was a positive CXR, significant bacterial count via PCR in induced sputum samples and either fever (≥ 38°) or neutrophil count ≥ 8.0 x 10^9/l.

Viral aetiology was presumed if they had a virus detected either via PCR, immunofluorescence or viral culture in induced sputum. Co-infection (bacteria and virus) aetiology would include children who had both a significant bacteria (as described above) and virus detected in the induced sputum.

Undefined aetiology was presumed if the above 3 criteria were not satisfied.

### Specimen collection and analysis

Once informed consent was obtained, induced sputum[11] with nebulised 3% saline and blood were taken within 24 hours of admission. Induced sputum samples to be tested for both virus and bacteria via PCR were immediately stored in -80°C freezer until further analysis. Induced sputum samples were also tested for viruses using immunofluorescence and culture. The 11 viruses investigated for were as follows: influenza A virus, influenza B virus, human metapneumovirus (hMPV), human parainfluenza virus types 1,2 and 3 (hPIV-1, hPIV-2, hPIV-3), human adenovirus (hAdV), human respiratory syncytial virus types A and B (hRSV-A, hRSV-B) human rhinovirus (hRV) and human bocavirus (hBoV). Blood and induced sputum were tested for six bacteria via PCR: *Staphylococcus aureus*, *Streptococcus pneumoniae*, *Haemophilus influenzae*, *Bordetella pertussis*, *Mycoplasma pneumoniae and Chlamydophila pneumoniae*. *Moraxella catarrhalis* was considered a significant pathogen if induced NPS culture had a pure growth of *Moraxella catarrhalis* in the presence of epithelial cell: pus cell < 1:10 [18,19].

Chest radiographs were reviewed by a designated radiologist who was blinded to the clinical presentation of the patient. The radiographic findings were reported according to the presence of alveolar, focal or diffuse infiltrates, air bronchograms, silhouette sign, hyperinflation, hilar lymphadenopathy, pleural effusion and atelectasis.

### Methodology for virus PCR

RNA from induced sputum samples was extracted using KingFisher^™^ Pure Viral NA Kit (Thermo Fisher Scientific, US). A modified multiplex nested PCR [20] was used to screen ten different respiratory viruses including influenza A virus, influenza B virus, hMPV, hPIV-1, hPIV-2, hPIV-3, hAdV, hRSV-A, hRSV-B, and hRV. In addition, PCR was used to screen for hBoV as previously described[21]. All PCR products were visualized by agarose gel electrophoresis.

### Methodology for bacterial PCR

Genomic DNA extraction was performed by a simple boiling method and the supernatant was used as the template for PCR assays. Confirmatory PCR screening targeting the transketolase (*RecP*) gene of S. *pneumoniae* and serotyping of S. *pneumoniae* using 30 serotype-specific primer pairs and internal control (*CpsA*) as described by Jourdain et al[22] were performed in a 25μl reaction mixture using a thermal cycler. The PCR products were electrophoresed on 1% (w/v) LE agarose (Promega, Madison, USA) gel containing SYBR® Safe DNA Gel Stain (Invitrogen Corporation, Carlsbad, CA, USA) and viewed under a UV transilluminator.

DNA extracted from ATCC was quantified to 100 ng/ml and ten-fold serial dilution was performed to achieve the concentration series from 10^11 to 10^3 copies. The calculation of copy number is based on the formula: copy number = (ng * number/mole) / (bp * ng/g * g/ mole of bp). Standard curves for quantification of all the organism were generated with the slope values from -3.48–3.67. Bacterial counts for organisms detected in induced sputum were considered significant if their copy number exceeded detection limit set by the standard curves: *S*. *pneumoniae* >6.7 log10/ml, *H*. *influenzae* >5.7 log10/ml, *S*. *aureus* >7.5 log10/ml, *M*. *pneumoniae* >6.6 log10/ml and *B*. *pertussis* >6.2 log10/ml. A pure growth of *M*. *catarrhalis*, in the presence of epithelial cell: pus cell < 1:10, was considered significant[18].

### Data collection

Data was acquired through face-to-face interviews and included socio-demographic data, birth and feeding history, personal and family history of asthma and atopy, environmental factors (out-of-home care, number of siblings etc), economic status including socioeconomic status measured by the number of appliances in the home, past medical history of respiratory illness, exposure to environmental tobacco smoke (ETS), vaccination history (which includes prior exposure to pneumococcal and/or influenza vaccines), clinical findings at admission, duration of admission, treatment received and baseline laboratory results (full blood count, renal function, blood culture and C-reactive protein[if taken])

Weight at admission was noted and changed into Z score by using gender and age specific cut- off points proposed by the World Health Organisation guidelines[23].

### Statistical analysis

Data analysis was performed using Statistical Package for Social Science (SPSS) software version 16.0 (IBM, USA). Continuous data was expressed as mean (standard deviation [SD]) if normally distributed or median (interquartile range [IQR]) if not normally distributed. Chi-square test was used for comparing categorical variables between two groups and odds ratio (OR) and 95% confidence interval (CI) were reported, where appropriate. Mann-Whitney U test was used when comparing continuous (numerical) variables without normal distribution between the two groups.

As the biggest clinical dilemma is the identification of bacterial pneumonia, we looked at factors associated with bacterial isolation: demographic factors (age in months, gender, duration of breast feeding, out-of-home care, number of children in the house, number of people in the house, number of people co-sleeping in the same room, educational level of parents, total household income, presence of pets, past history of lower respiratory tract infection, ETS exposure, pneumococcal vaccination, antibiotic use) clinical complaints (noisy breathing, diarrhoea, vomiting,) examination findings (weight according to Z-score, highest recorded temperature, wheeze, crepitations). As for associations between co-infection and severity of clinical illness, the following factors were entered into univariate analysis: need for oxygen, non-invasive ventilation, intravenous infusion, nasogastric tube feeding, duration of hospitalisation. Investigation findings (neutrophil count and chest radiograph [CXR]) were not included in the model as these were used to define bacterial infection.

Bivariate logistic regression was used to determine significant factors associated with bacterial isolation and severity of infections as well as associations between co-infections and markers of a clinically severe illness. Factors in univariate analysis that had a p<0.10 were entered in logistic regression (Enter method). All tests were calculated in a two-tailed manner and significance was defined by a p value of less than 0.05.

## Results

**Fig 1:**
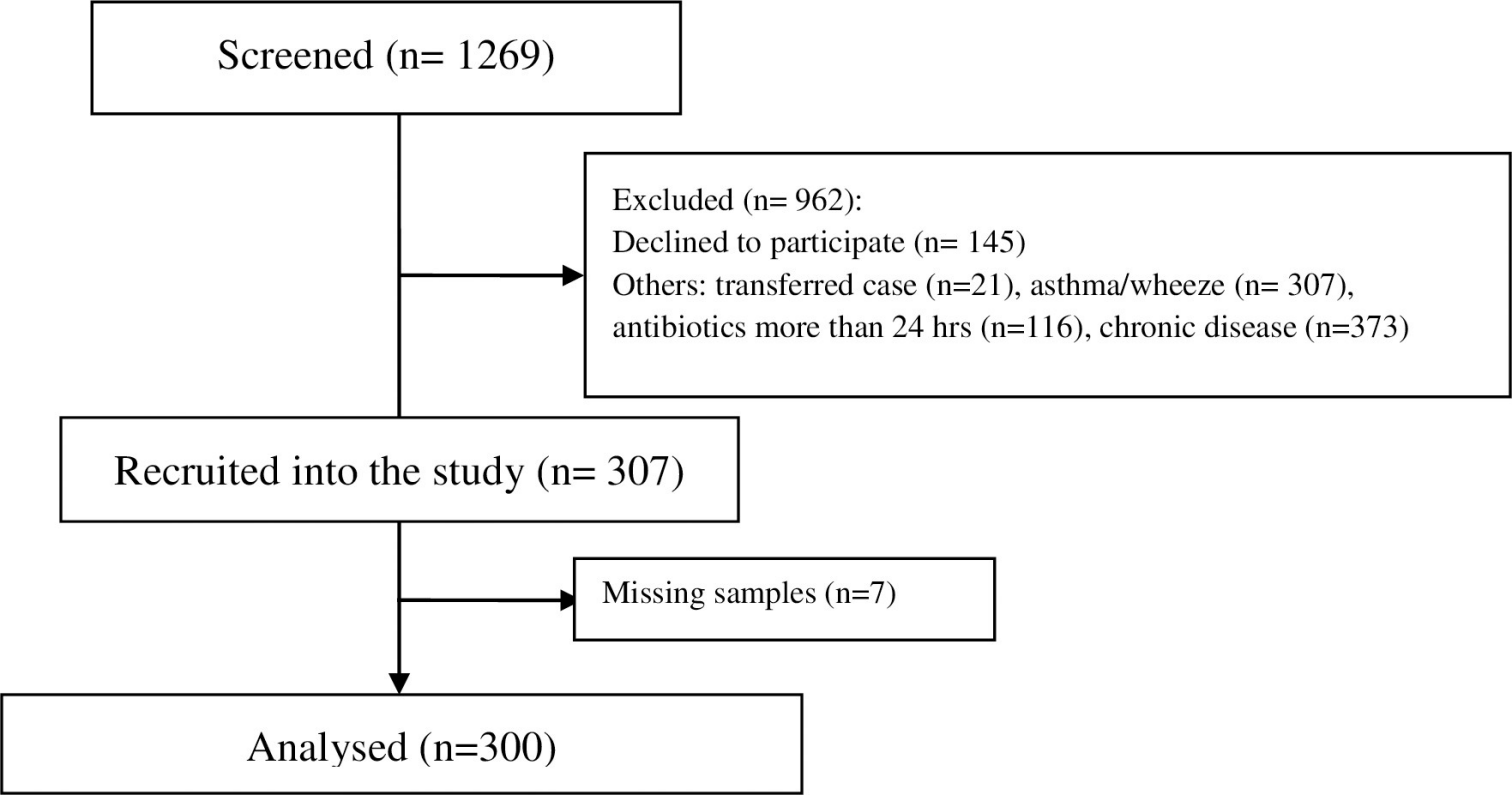
CONSORT flow diagram.

A total of 307 patients were recruited between 1^st^ October 2014 and 31^st^ October 2015; however, there were 7 missing samples and 300 specimens were finally analysed. Study flow is shown in the CONSORT flow diagram (Fig 1).

### Demographic data

The mean (SD) age of the patients was 14 (± 15) months old, with age range from 1–137 and there was a male predominance (n = 184, 61%). The rest of the demographic data is shown in Table 1. Only half of the parents (n = 151, 50.3%) had heard of the pneumococcal vaccine.

**Table 1:** Demographic characteristics of the patients.

Demographic characteristics	N	%
**Age, mean (SD) months**	14 (15)	-
**Gender**		
Male	184	61
Female	116	39
**Ethnicity**		
Malay	263	87.7
Chinese	11	3.7
Indian	21	7
**Breast fed ≥ 6mths**	116	38.7
**In out-of-home care**	192	
**Number of children in household, median (IQR)**	5(4,6)	-
**Number of people in household, median (IQR)**	3(3,4)	-
**Number of people co-sleeping with child, median (IQR)**	3 (3,4)	-
**Father’s education**^**a**^		
Low	216	72
High	84	28
**Mother’s education**^**a**^		
Low	200	66.7
High	100	33.3
**Total income,** **Median (IQR)**		
**Has a past history of lower respiratory tract infection**	70	23
**Exposed to ETS**	143	47.7
**Vaccinated against pneumococcal**	26	8.7
**Vaccinated against Hib**	270	90.0
**Vaccinated against Influenza**	11	3.7
**Weight z score, median (IQR)**	-1.07 (-5.54 to 4.00).	-
**Prior antibiotics before admission**	87	29

^a^Low education is defined as low if have diploma and less while high was defined higher than diploma

Pathogens were detected in two-thirds of patients (n = 186, 62%) while in a third of patients (n = 114, 38%), neither bacteria nor virus were isolated, despite these rigorous microbiological methods.

### Bacteria detected in induced sputum and blood samples via PCR

In nearly half of the patients (n = 139, 46.3%), induced sputum had bacteria isolated via PCR: 65.4% (n = 91/139) as bacteria alone and 33.8% (n = 47/139) together with a virus. The commonest bacteria (alone or with other bacteria/virus) isolated were *H*. *influenzae* (n = 57), *S*. *aureus* (n = 56) and *S*. *pneumoniae* (n = 37) and while *M*. *pneumoniae* (n = 1) and *B*. *pertussis* (n = 2) were rarely detected. All *H*. *influenzae* strains were *non-typeable Haemophilus*.*influenzae (*NTHi*)*. None of the children were positive (via PCR) for *C*. *pneumoniae*. Induced sputum cultures were positive (pure growths) for *M*. *catarrhalis* in 2.9% of specimens (n = 4). **S1 Table**

As for organisms detected via PCR in blood samples, only *S*. *aureus* (n = 4) was detected. In one of these patients only the blood PCR was positive for *S*. *aureus* while the induced sputum was positive for both hMPV and rhinovirus. In the one patient who was *M*. *pneumoniae* positive, PCR was positive in both blood and induced sputum. In 19 patients (13.7%), more than 1 bacteria were detected via PCR.

Blood cultures were positive for 3 (1%) children: *H*. *influenzae (n = 1)*, *S*. *pneumoniae (n = 1)* and *S*. *aureus (n = 1)*.

### Definite bacterial pneumonia or bacterial infection

**Fig 2:**
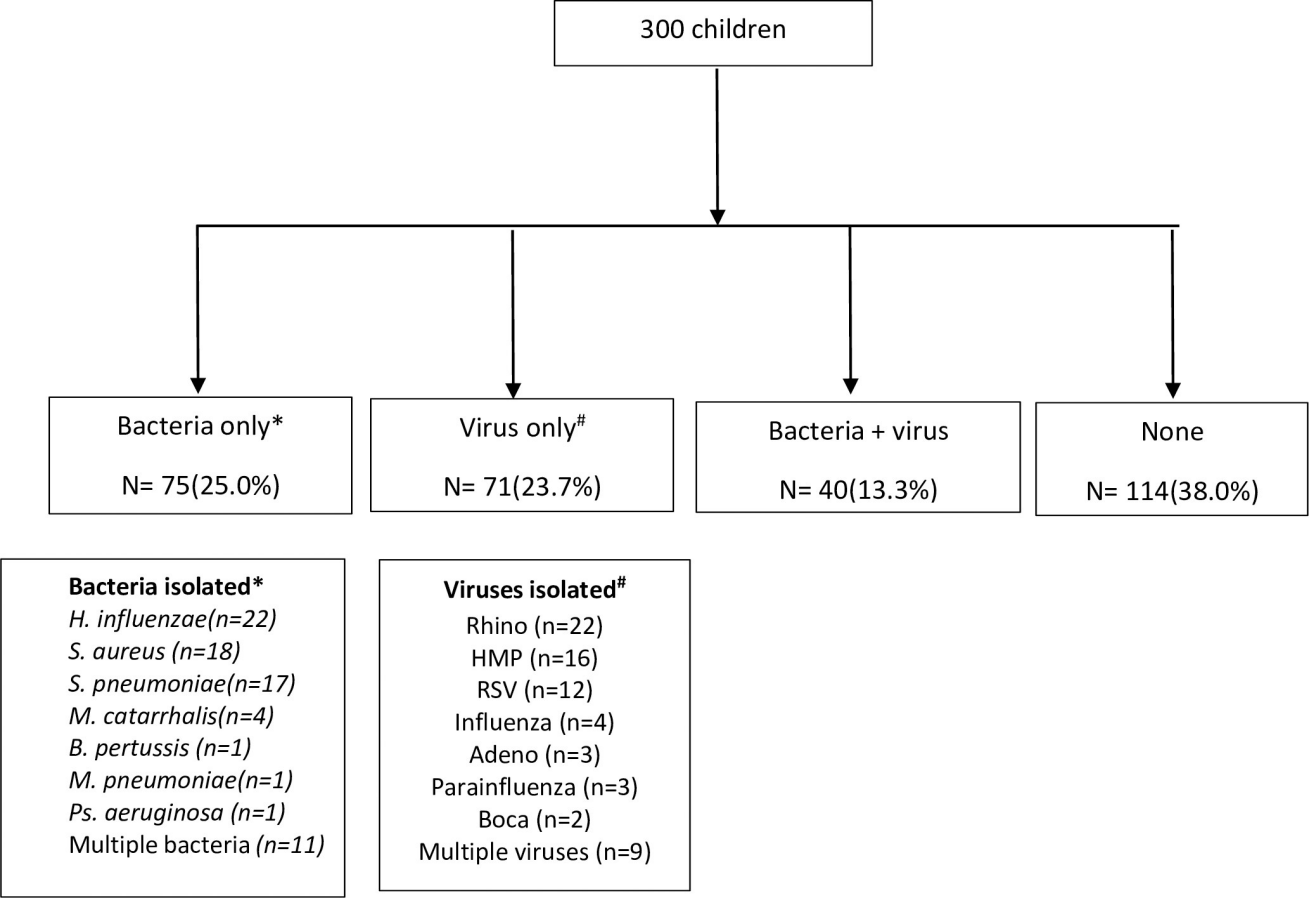
Shows the significant organisms detected in these children, after separating into those with bacteria alone, virus alone and co-infections (bacteria and virus together).

As mentioned above, definite bacterial infection pneumonia was presumed if there was a positive CXR, significant bacterial count and either fever or neutrophil count ≥ 8.0 x 10^9/l. [17] Using this definition, 75 (25.0%) children had a definite bacterial pneumonia, and 40 (13.30%) had a co-infection. Bacteria implicated were as follows: NTHi (n = 22, 29.3%), *S*. *aureus* (n = 18, 24.0%), *S*. *pneumoniae* (n = 17, 22.7%), *M*. *catarrhalis* (n = 4, 5.3%), multiple bacteria (n = 11, 14.7%) with *B*. *pertussis* (n = 1, 1.3%), *M*. *pneumoniae* (n = 1, 1.3%) and *Pseudomonas aeruginosa* (n = 1, 1.3%). (Fig 2)

### Viruses detected in children admitted with LRTIs (Fig 2)

As for viruses, more than a third of patients (n = 111, 37.0%) had viruses detected in their induced sputum: virus alone detected in 23.7% (n = 71) and virus together with bacteria in 13% (n = 40). Viruses isolated alone were rhinovirus (n = 22, 31.0%), RSV (n = 12, 16.9%), hMPV (n = 16, 22.5%), influenza (n = 4, 5.6%), parainfluenza (n = 3, 4.2%), adenovirus (n = 3, 4.2%), bocavirus (n = 2, 2.8%) and multiple viruses (n = 9,12.7%).

Neither RSV nor hMPV was associated with increased duration of hospitalisation (z = -0.63, p = 0.50) and (z = -0.34, p = 0.74) respectively or life-threatening infection (OR 0.31[95% CI 0.07,1.36], p = 0.10) and (OR 2.38 [95% CI 0.98,5.80], p = 0.06) respectively.

### Virus + bacteria detected (Table 2)

Co-infection (virus + bacteria) was seen in 13.3% (n = 40) of patients. Commonest viruses isolated were RSV (n = 15, 37.5%), rhinovirus (n = 10, 25.0%) and hMPV (n = 6, 15.0%) and significant bacteria were NTHi (n = 16, 40.0%), *S*. *aureus* (n = 9, 22.5%) and *S*. *pneumoniae* (n = 7, 17.5%).

**Table 2:** Co-infections i.e. bacteria and virus isolated in children admitted with very severe lower respiratory tract infection.

Organisms	NTHi^a^	S. aureus	S. pneumoniae	M. catarrhalis	B. pertussis	2 or more bacteria	Total
**RSV**	3	5	4	0	1	1	**15**
**Rhinovirus**	4	1	1	0	0	4	**10**
**hMPV**	5	0	1	0	0	0	**6**
**Parainfluenza**	0	0	0	0	0	0	**0**
**Influenza**	0	0	0	0	0	0	**0**
**Adeno**	2	0	0	0	0	0	**2**
**Bocavirus**	0	0	0	0	0	1	**1**
**2 or more** **viruses**	2	2	1	0	0	1	**6**
**Total**	**16**	**9**	**7**	**0**	**1**	**7**	**40**

^a^NTHi = non-typeable *Haemophilus influenzae*

### Factors associated with bacterial pneumonia (Table 3)

Factors looked at for association with bacterial isolation are shown in Table 3. In both univariate and bivariate logistic regression, male gender (AdjOR 1.84) and presence of crepitations (AdjOR 2.27) were independently associated with bacterial pneumonia.

**Table 3:** Demographic and clinical factors that could be associated with significant bacterial detection (bacteria only or co-infections).

Factors		Bacteria Yes N = 115 (%)		Bacteria No N = 185 (%)	Crude OR/ Z score	95% CI	P value	Adj OR	95% CI	P value
**Age, median (IQR) months**		9(5,17)		10(6,17)	Z = -.68		0.50			
**Gender**^**a**^										
Male		80(70)		104(56)	**1.78**	**1.09,2.91**	**0.02**	**1.84**	**1.10,3.05**	**0.02**
Female		35(30)		81(34)						
**Duration of breast feeding, median (IQR) months**		3(2,7)		4(2,6)	Z = -.49		0.63			
**BF ≥ 6mths**										
Yes		69(60)		114(62)	0.96	0.59,1.54	0.85			
No		45(39)		71(38)						
**Our of home care**										
Yes		72(63)		120(65)	0.92	0.56,1.52	0.75			
No		39(34)		60(32)						
**Number of children in household, median (IQR)**		2(2,3)		2(1,3)	Z = -.53		0.60			
**Number of people in household, median (IQR)**		5(4,6)		5(4,6)	Z = -.09		0.93			
**Number of people co-sleeping with child, median (IQR)**		3(3,4)		3(3,4)	Z = -.09		0.99			
**Father’s education**^**b**^										
Low		88(77)		128(69)	1.45	0.85,2.47	0.17			
High		27(23)		57(31)						
**Mother’s education**^**b**^										
Low		81(70)		119(64)	1.32	0.80,2.18	0.28			
High		34(30)		66(36)						
**Total income,** **Median (IQR)**		5000 (3400,7000)		5000 (3053,6900)	Z = -.68		0.50			
**Pets**										
Yes		19(17)		22(12)	1.44	0.74,2.81	0.28			
No		97(84)		162(88)						
**Past history of lower respiratory tract infection**										
Yes		30(26)		40(22)	1.28	0.74,2,20	0.38			
No		85(74)		145(78)						
**ETS exposure**										
Yes		57(50)		93(50)	0.97	0.61,1.55	0.91			
No		58(50)		92(50)						
**Vaccination against pneumococcal**										
Yes		8(7)		18(10)	0.69	0.29,1.65	0.40			
No		106(92)		165(89)						
**Prior antibiotic use**										
Yes		31(27)		56(30)	0.85	0.50,1.43	0.53			
No		78(67)		119(65)						
Clinical Parameters										
**Weight z score, median (IQR)**		-1.07 (-1.74,0.16)		-1.07 (-2.08,0.26)	Z = -.70		0.48			
**Noisy breathing** ^**a**^^,^^**c**^										
Yes		69(60)		91(49)	**1.58**	**0.99,2.54**	**0.06**	**1.45**	**0.88,2.37**	**0.14**
No		45(39)		94(51)						
**Vomiting a**,^**c**^										
Yes		56(49)		71(38)	**1.55**	**0.97,2.85**	**0.07**	**1.56**	**0.96,2.54**	**0.08**
No		58(50)		114(62)						
**Diarrhoea** ^**c**^										
Yes		20(17)		30(16)	1.09	0.59,2.05	0.77			
No		95(82)		154(84)						
**Highest temperature**		38.7(38,39)		38.1(37.9,39)	Z = -1.27		0.19			
**Wheeze**										
Yes		44(38)		87(47)	0.69	0.43,1.12	0.14			
No		69(60)		75(41)						
**Crepitation**^**a**^										
Yes		102(89)		143(77)	**2.30**	**1.15,4.60**	**0.02**	**2.27**	**1.12,4.70**	**0.02**
No		12(10)		39 (21)						
**CRP mg/l, median (IQR)**		1.6 (0.6,5.1)		1.5 (0.6,3.5)	Z = -1.01		0.31			

^a^Significant in univariate analyses and hence entered into bivariate regression analyses;

^b^low educational level is up to diploma and high educational level is from university and above;

^c^symptoms

### Co-infection and clinical severity of illness

When looking at the association between co-infections and clinical severity of illness, the CRP (z = -2.69, p = 0.007) and initial heart rate (z = -2.54, p = 0.01) were significantly higher in co-infections. However, other clinical indicators of severity of illness were not associated with co-infection: age in months (z = -0.35, p = 0.73), need for oxygen (p = 0.69, OR 1.16 [95%CI 0.56, 2.39]), need for nasogastric tube feeding (p = 0.60, OR 1.16 [95%CI 1.11, 1.22]), need for IV fluids (p = 0.16, OR 1.62[95% CI 0.83, 3.16]) need for ventilation (non-invasive and invasive) [p = 0.27, OR 1.59[95% CI 0.67, 3.54] and duration of hospitalisation (z = -0.29, p = 0.77).

## Discussion

This study set out to determine the pathogens isolated in children admitted with very severe lower respiratory tract infections, from a developing country. Pathogens were detected in two thirds of all children, with about one in four children either having a bacteria alone or a virus alone and 13% having both a virus and bacteria (co-infection). Bacterial pneumonia was seen more in males and when there were examination findings of crepitation. In this study, patients with co- infection had a higher CRP and initial heart rate but no other markers of clinical severity like longer duration of hospitalisation and need for increased respiratory or medical support.

Ascribing aetiology of pneumonia is very difficult. Many aetiological studies have avoided directly associating detection of bacteria with disease, due to the high nasal carriage rates of bacteria in children compounded by the inability of children to produce high quality sputum, uncontaminated by upper airway “colonisers”[24]. In this study, steps were taken to ensure that the pathogen isolated would be significant, especially for bacteria, as this was our main interest. We used both blood and induced sputum to determine significant pathogens[19]. Blood and induced sputum were also taken within 24hrs of admission to avoid nosocomial contamination and eradication with antibiotics. We excluded the wheezing phenotype as the main reason for admission would be their predisposition to wheezing causing the respiratory distress rather than the organism’s pathogenicity. Finally, interpretation of a positive bacterial infection was done using copy numbers and positive cut offs determined by standard curves[25]. Final positive copy numbers used in this study were similar to that found in the PERCH study, except for *S*. *aureus*. In this study, the cut off for *S*. *aureus* was much higher than in the PERCH study (which was 3.0 log10/ml) and the Drakenstein Child Health Study (median [IQR] of 5.12[3.71–6.42] found in controls). In this study we used *S*. *aureus* >7.5 log10/ml as per our standard curves[26,27]. This should be considered a strong point for this study as *S*. *aureus* is often considered a coloniser, yet we now know that colonisers, given the right circumstances and environment, can invade the host and cause disease[28–30]. Studies have also shown that detection of bacteria and higher colony counts of *S*. *pneumoniae*, *M*. *catarrhalis* and *H*. *influenzae* were associated with clinical febrile pneumonia in children[26,27,31].

In this study, we detected significant pathogens in about two-thirds of patients, similar to some studies[3,32] but lower compared to others that report pathogen detection of up to 98.6% of cases[33]. In a third of pneumonia cases, no aetiology was detected, which could be due to the exclusion of recurrent wheezers as well as by our very strict definition of bacterial pneumonia. In a very large study in India published in 2015, multiplex PCR detected pathogens in 98.6% of the 428 samples tested with 82.2% having multiple organisms[34]. In a study from Kenya, where they used paired serology (acute and convalescent) for five viruses and *B*. *pertussis* and a 15 virus PCR kit whereas bacteria was detected from NPA cultures, bacterial detection was low at 9% and viral detection high at 53%. The prevalence of co-infection was 15%, similar to our study. Therefore, methods used to detect pathogens will affect the detection rate. In our study, the multiplex PCR used detected 16 viruses/bacteria while in other studies, it detected 25 viruses/bacteria[34]. While extensive PCR panels improve the detection rate, it may be oversensitive thus making it difficult to determine which of the detected organisms is the offending agent, especially when many organisms are detected in one patient as in studies where as much as 82% had multiple organisms[34][35]. In this study, we included only children with very severe pneumonia and did both bacterial and viral PCR on induced sputum and blood, hoping to increase the sensitivity and specificity of the test. The number of multiple detections (bacteria + bacteria [n = 11]) and virus + virus [n = 9]) were relatively low compared to other studies[25,33,34].

We defined bacterial pneumonia as children presenting with very severe pneumonia with a positive CXR and having either a fever or a high neutrophil count. Here we found that 25% had a definite bacterial infection. Overall, NTHi was the commonest bacteria implicated followed by *S*. *aureus*, while *S*. *pneumoniae* was the third commonest. The low prevalence of *S*. *pneumoniae* in our study is an interesting finding as pneumococcal vaccination is not part of the national immunisation programme and since only 8% of the patients in this study had received at least one dose of the pneumococcal vaccine, vaccination alone cannot explain this low prevalence. This may reflect the aetiology of pneumonia in a developing country. In most studies, pneumococcal infection is the commonest bacterial aetiology[3,34] however *H*.*influenzae* has increasingly been recognised as an important cause of respiratory tract infection in children including pneumonia[36–38]. In the Drakenstein Child Health Study in Africa, where pneumococcal vaccination is included in their national immunization programme, they found a strong association between RSV, *B*. *pertussis* and *H*. *influenzae* with pneumonia, and that *H*. *Influenzae* occurred most frequently[25]. In a review article by Zar et al, the authors highlighted the changing disease spectrum of pneumonia in childhood, particularly the reducing prevalence of *S*. *pneumoniae* and Hib amid widespread use of vaccination[39]. In a large prospective study in India, *S*. *aureus* was detected in about 7% of children admitted with a LRTI. Similarly, in our study, *S*. *aureus* was *isolated* in 9% of all children[34]. None of the isolates in our study were *Methicillin -resistant S*. *aureus*.

We also set out to determine the predictors of bacterial infection and found that male sex and presence of crepitations were independent predictors, with signs of crepitation being the strongest predictor. Crepitations have been shown to be associated with pneumonia[40]. Male children have been shown to have an increased risk for respiratory infections, which would also include bacterial infections[41].

In this study, viruses were isolated in 37% of all patients with the commonest being rhinovirus, RSV and hMPV. This detection rate is lower compared to others who have detected viruses in 60% to 98% of cases with LRTI[3,25,33,34]. This could be explained by the fact that our PCR did not detect enteroviruses as did other studies as well as the exclusion of recurrent wheezers who are usually infected by viruses[34]. While both RSV and rhinovirus are commonly seen all over the world [25,33,34] it is interesting that hMPV was the 3^rd^ commonest virus in our study[35]. hMPV is a cause of severe respiratory tract infection and symptoms are usually indistinguishable from RSV[42,43]. hMPV is reported to be detected in 4–16% of patients with respiratory tract infection, which makes it a significant virus [35,44,45]. In this present study, it was detected in 7% of all patients admitted with LRTI. However, neither RSV nor hMPV was associated with serious infection, when looking at duration of hospitalisation and need for increased respiratory support.

In this study, co-infections were seen in 13% of patients. The commonest viruses detected were rhinovirus and respiratory syncytial virus while the commonest bacteria were *H*. *influenzae* and *S*. *aureus*. Our findings concur with those from Kenya and the UK where co-infections were found in 15% and 12.5% of cases respectively[3,32] but others have found prevalence of mixed infections as high as 82%. [34,46]. Co-infections have been associated with severe clinical illness in some studies, but not all, with a recent metanalysis finding no significant association with markers of severe illness[47]. In this study, co-infections were associated with a higher CRP and presenting heart rate but not other significant markers of severe illness i.e. longer duration of hospitalisation, need for NIV, PICU care, oxygen therapy and nasogastric tube feeding. The CRP being higher in co-infection has been shown before[48].

Atypical bacteria like *M*. *pneumoniae* and *B*. *pertussis* were extremely rare in our population and *C*. *pneumoniae* was not detected in this study. As we know *M*. *pneumoniae* and *C*. *pneumoniae* are usually seen in children older than 5 years[49] while our study population had a mean age of 14 months. *B*. *pertussis* was rarely detected probably as the majority of our patients (90%) had had their scheduled vaccinations. Our finding of only 3% of children from our study being positive for atypical organisms was similar to a previous Asian study using paired serology in children more than 2-years old for *M*. *pneumoniae*, *C*. *pneumoniae and Legionella pneumophila*[50].

Limitations in this study are recognised. We did not determine the prevalence of organisms detected via PCR in a control group of healthy children as studies have shown a high number of organisms in these children, probably the result of overlapping viral shedding and bacterial colonisation[25]. Inclusion of control children would have reduced the over-detection of non-pathogenic organisms especially viruses. However, case-control studies have shown that RSV is the main pathogen [3] and in this study, we found that both RSV and hMPV were commonly isolated, and these are unlikely to be innocent bystanders[3,25,51]. We performed multiplex PCR on blood and induced sputum, induced sputum viral and bacterial culture and IF for viruses and blood culture to detect both viruses and bacteria. However, urine antigen test for pneumococcal infection and convalescent serology tests to detect atypical bacteria and viruses were not done. This may result in underestimation of the burden of bacteria and viruses in LRTI. However, urine antigen tests lack specificity in children due to the high nasal carriage of *S*. *pneumoniae* [52,53] and serology testing is complex, even in a clinical scenario, and hence not used in this study. Finally, this study was carried out in an urban population and hence the aetiology may not be generalised to the whole population.

Currently there is no gold standard test that is adequately sensitive and specific to determine the aetiology of pneumonia with certainty. There is also a dire need for a point-of-care test in determining aetiology of lower respiratory tract infections. Although viruses are commonly detected, and usually initiate the respiratory infection, secondary bacterial infection may occur, and hence with-holding antibiotics when a virus is detected may not be wise[13].

## Conclusion

Significant pathogens were detected in two-thirds of all children with one in four children admitted for very severe LRTI having bacteria alone, one in four children having a virus alone and 13% having a mixed infection. Males and presence of crepitations were significantly associated with bacterial infection. Co-infection was associated with a higher CRP and heart rate but not with other parameters of severe infection. Although viruses are commonly detected, and usually initiate the respiratory infection, secondary bacterial infection may occur, and hence with-holding antibiotics when a virus is detected may not be wise. ^13^

## Supporting information

S1 Table Summary of the bacteria detected via PCR in induced sputum of children admitted for very severe pneumonia. ^a^ In 19 patients more than 1 bacteria were detected via PCR in induced sputum.
(PDF)
