# Supplementary material for: Bacterial pneumonia and its associated factors in children from a developing country: A prospective cohort study
Source: PLoS One. 2020 Feb 14;15(2):e0228056. doi: 10.1371/journal.pone.0228056 (PMC7021284; doi:10.1371/journal.pone.0228056)
Supplement: S1 Table — a In 19 patients more than 1 bacteria were detected via PCR in induced sputum. (PDF) [file pone.0228056.s001.pdf]

S1 Table : Summary of the bacteria detected via PCR in induced sputum of children admitted for very severe pneumonia.

**Bacteria Detected[a]**	**N = 139**<br>**N**	**%**
H. influenzae	57	41
S. pneumoniae	37	26.6
S. aureus	56	40.3
M. catarrhalis	4	2.9
B. pertussis	2	1.4
M. pneumoniae	1	0,7
Chlamydophila pneumoniae	0	0

[a] In 19 patients more than 1 bacteria were detected via PCR in the induced sputum
